# Removal of substrate inhibition of *Acinetobacter baumannii* xanthine oxidase by point mutation at Gln-201 enables efficient reduction of purine content in fish sauce

**DOI:** 10.1016/j.fochx.2023.100593

**Published:** 2023-02-04

**Authors:** You Wen, Jiahui Xu, Donglei Pan, Chenghua Wang

**Affiliations:** College of Light Industry and Food Engineering, Guangxi University, Nanning 530004, People’s Republic of China

**Keywords:** Substrate inhibition, *Acinetobacter baumannii*, Xanthine oxidase, Fish sauce, Point mutation, Enzyme engineering

## Abstract

Xanthine oxidase is an oxidase that has a molybdopterin structure with substrate inhibition. Here, we show that a single point mutation (Q201) in the *Acinetobacter baumannii* xanthine oxidase (AbXOD) obtained mutant Q201E (*k*_cat_ =799.44 s^−1^, no inhibition) with high enzyme activity and decrease of substrate inhibition in 5 mmol/L high substrate model, and which cause two loops structure change at active center, characterized by complete loss of substrate inhibition without reduction of enzymatic activity. Molecular docking results showed that the change of flexible loop increased the affinity between substrate and enzyme, and the formation of a π-π bond and two hydrogen bonds made the substrate more stable in the active center. Ultimately, Q201E can still maintain better enzyme activity under high purine content (an approximately 7-fold improvement over the wild-type), indicating a broader application prospect in the manufacture of low-purine food.

## Highlights

• Single point mutation removes substrate inhibition of *Acinetobacter baumannii* XOD.
• XOD was firstly used to degrade xanthine in high-purine-content fish sauce.
• Q201E completely lost substrate inhibition and nearly 2-fold improved enzymatic activity.
• Q201E produced 7-fold uric acid, and 14.6-fold xanthine to the WT in fish sauce.
• Conformation changes of two flexible loops might underline the SI removal in Q201E.

## Introduction

Xanthine oxidase (XOD, EC 1.17.3.2) is a flavoprotein with a molybdopterin structure that can use molecular oxygen as electron acceptor to catalyze the degradation of hypoxanthine into uric acid in two steps (Bortolotti et al., 2021, Cao et al., 2010). XOD not only plays a key role in purine metabolism, but also can be used as a target for the development of anti-gout drugs like allopurinol and febuxostat, as a model enzyme for the mechanism study of free radical molecular damage, and purine metabolizing enzymes in the precision manufacturing of low-purine foods. However, previous studies have found that XOD is subjected to obvious Substrate inhibition (SI) (Cao et al., 2014, Hausladen and Fridovich, 1993, Rubbo et al., 1991), which limits the XOD activity and its development and application expansion under high substrate concentrations.

Purines are heterocyclic aromatic compounds that occur ubiquitously in all living organisms throughout nature and the main cause of gout (Colette Daubner and Lanzas, 2018, Foucher and Faure, 2022). According to the previous investigations, seafood products have high contents of xanthine and hypoxanthine (Kaneko, Aoyagi, Fukuuchi, Inazawa, & Yamaoka, 2014). For example, xanthine in oysters can reach 82.1 mg/100 g, in oriental shrimp can reach 117.1 mg/100 g, and hypoxanthine in fresh marine fishes can generally reach 100 mg/100 g. Jankowska et al. made the first attempt to use purine metabolic pathway enzymes as additives for purine degradation to produce low-purine foods by overexpressing purine metabolic enzymes from *Arxula adeninivorans* (Jankowska et al., 2015, Trautwein-Schult et al., 2013). Mahor et al. found that the application of a mutant purine nucleoside phosphorylase, KlacPNP N256D from *Kluyveromyces lactate* reduced the purine content in beer (Mahor, Priyanka, Prasad, & Thakur, 2016). Fish sauce is a clarified seasoning made by the natural fermentation of marine fish (Endo et al., 2014, Kuda, 2015). Through sampling investigation and high-performance liquid chromatography (HPLC) determination, we found that the content of hypoxanthine in commercial fish sauce seasoning can reach 2-3 mmol/L (unpublished data), which is far higher than the substrate concentration used for assaying XODs in previous studies that are not exceeding 0.2 mmol/L (Kostić, Dimitrijević, Stojanović, Palić, Đorđević, & Ickovski, 2015). So, there is a need to develop new mutant XODs, which are not subjected to SI for effective degradation of hypoxanthine content in fish sauce to produce nutritional low-purine food catering to hyperuricemia and gout patients.

SI is a common phenomenon existing in about a quarter of known enzymes in the process of industrialization (Reed et al., 2010, Wu, 2011, Yoshino and Murakami, 2015). Although SI has vital physiological and pharmacological functions, it is unserviceable in industrial applications of enzymatic reactions. Some enzymes have been reported to cease working because of SI under relatively high substrate concentrations, severely affecting enzymatic productivity (Shang et al., 2020, Torrelo et al., 2014). To solve this problem, two kinds of protein engineering approaches have been developed: one is reducing substrate affinity and relieving substrate inhibition through modification of relevant amino acids in the enzyme active center, and the other is improving substrate tolerance and increasing enzymatic activity through altering residues outside active site including allosteric sites to indirectly optimize the substrate binding and product release (Kokkonen et al., 2021, Shang et al., 2020, Torrelo et al., 2014).

Xanthine dehydrogenase (XDH, EC 1.17.1.4) and XOD are highly related single-gene products that exist in separate but interconvertible forms, of which the XDH can be converted to XOD reversibly by oxidation of cysteine residues or irreversibly by limited proteolysis (Nishino et al., 2005). In our previous work, we reported a system for the heterologous expression of *A. baumannii* XDH in *Escherichia coli* (C. H. Wang, Zhao, Li, Zhang, & Xing, 2016), in which we compared the crystal structure of bovine milk XDH (PDB ID: 3EUB) with a homology model of the *A. baumannii* enzyme which was constructed using the Swiss-model server with the *R. capsulatus* XDH coordinates as a template. One major difference between the bovine XDH (BtXDH) and *A. baumannii* XDH (AbXDH) is the replacement of Thr1010, Val1011, and Pro1012 with Asn458, Ala459 and Val460 in the substrate-binding pocket of *A. baumannii* XDH (Cao et al., 2010, Cao et al., 2014, Pauff et al., 2008). Through protein engineering on the A subunit of AbXDH, our group created a mutant, Y351V, by substituting the tyrosine at 351 to valine, which converts the AbXDH into an XOD (AbXOD, unpublished). The mutant Y351V showed maximum oxidase activity at pH 7.0 and 50 ℃ with a *k*_cat_ value of 409.65 s^−1^.

Taking into consideration that targeted mutation of key amino acid residues on the substrate channel and binding pocket can relieve substrate inhibition (Kokkonen et al., 2021), in order to obtain mutable key sites, a structure-based multiple sequence alignment of the *A. baumannii* XOD and other 18 XOD family members was built to analyze the amino acid conservation pattern (Robert & Gouet, 2014). As shown in Supplementary Fig. 1, the sites 200-201 of B subunit of AbXOD located near the substrate channel are of the highly conserved α-helix with varying residues. There are 6 naturally occurring amino acids, namely Ala (5/19), Asp (5/19), Asn (4/19), Gly (3/19), Glu (1/19), Ser (1/19) which appear in 200^th^ site of B subunit (numbering in AbXOD), while the 201^st^ residue is relatively more conserved, the majority of which is Gly (12/19) and then comes Gln (2/18), Asp (1/19), Ala (1/19), His (1/19), Thr (1/19) and Glu (1/19). In this study, we carried out site-saturation mutagenesis at Q201site of AbXOD, then screened out beneficial mutations alleviating SI under high substrate concentrations, then studied the contribution of Q201 to the inhibition mechanism and application of mutants in decreasing purine contents in fish sauce.

## Materials and methods

### Bacterial strains and plasmids

*Escherichia coli* DH5α and pTrc99A were used respectively as host strain and expression vector for the expression of wild-type AbXOD and mutants as previously described (C. H. Wang et al., 2016).

### Site-directed mutagenesis and selection

Single point mutations at Q201 site were realized by PCR amplification using a pair of degenerate primers: forward primer (5’-GATTCCACAAGAAAATNNKAGTTTAAAAGTCTATTGCTCGACACAACAT-3’) and reverse primer (5’-GCAATAGACTTTTAAACTMNNATTTTCTTGTGGAATCGCATAAGAAATC-3’), with plasmid pTrc99a-Y351V as template. Primer STAR Max DNA Polymerase from Takara Company was used for PCR amplification and the demethylase DMT (Trans Inc.) was used to remove the methylated DNA template in PCR products. The demethylated PCR product was transformed into chemically competent *E. coli* DH5α cells to create the mutants. The mutants were screened by assaying their activities under a high substrate concentration of 5 mmol/L, and those showing greater enzyme activity than the wild type AbXOD were selected and further confirmed by DNA sequencing (BGI, Beijing, China).

### Protein expression and purification

The plasmid-bearing strains were cultured in Luria–Bertani broth (LB) medium containing ampicillin (50 μg/mL) at 37℃, 220 rpm. when OD600 of the culture reached 0.6, a final concentration of 0.25 mmol/L isopropyl1-thio-β-D-galactopyranoside (IPTG) was added and incubated at 30℃, 200 rpm for 18 h to induce the overexpression of recombinant proteins. The induced cells were harvested by centrifugation at 8000 rpm for 10 min. The cell pellets were suspended in 30 mL equilibration buffer (50 mmol/L phosphate buffer pH 7.5 containing 0.1% Triton X-100) and disrupted by sonication with a SCIENTZ JY92-IIN ultrasonic homogenizer (SCIENTZ, China) in 30 min. Disrupted cells were centrifuged at 12000 rpm and 4℃ for 50 min (Himac, Japan). The crude extract was collected, filtered, and loaded onto a Ni-NTA Superflow Cartridge (Cytiva, Sweden) in equilibration buffer (C. H. Wang et al., 2016). Unbound and weakly bound proteins were washed out using increasing imidazole concentrations. The target enzyme was then eluted with purification buffer containing 100 mmol/L imidazole. Eluted enzyme solution was concentrated at 1.5 mL using a 10 K ultrafiltration tube (Millipore). The purity of the protein was checked by SDS-polyacrylamide gel electrophoresis (SDS-PAGE). Protein concentrations were determined using BCA method with bovine BSA as standard (Smith et al., 1985).

### Enzyme assays

The XOD activity was investigated at two different concentrations of xanthine, 1 mmol/L and 5 mmol/L xanthine, by measuring the formation of uric acid using 100 mmol/L Tris/HCl buffer (pH 8.5), 1 mmol/L EDTA, 0.1 mmol/L Potassium Oxonate at 40℃.

The formation of uric acid was measured at 314 nm with a molar absorption coefficient of 39.2 mmol^−1^L^−1^ cm^−1^. One unit of XOD activity was defined as the amount of enzyme that forms 1 µmol uric acid in 1 min under the assay conditions.

### Enzyme characterization

The effect of pH on XOD activity was investigated at 40 ℃ in 50 mmol/L sodium phosphate (pH 5.5–9), Tris/HCl (pH 8.5-9.5), sodium carbonate buffers (pH 9-10.5), and Sodium Phosphate-NaOH (10.5-12). The pH-dependent stability was measured by incubating XOD (0.05 mg/mL) in 50 mmol/L of buffer (pH 5.5–12) for 14 h at 4 ℃ prior to measurement of the residual enzymatic activity. The effect of temperature on XOD activity was examined in 50 mmol/L Tris/HCl buffer (pH 8.5) at temperatures between 30 and 80 ℃. Thermal stability was determined by the residual activity after incubating XOD at temperatures ranging from 30 to 80 ℃ for 30 min.

The kinetic characterization of recombinant enzymes was carried out using 10 µg/mL purified XOD and varying concentrations of substrates (0.005–8 mmol/L) in 50 mmol/L Tris/HCl buffer (pH 8.5) at 40 ℃. The apparent Michaelis constant (*K*_m_) and turnover number (*k*_cat_) were deduced from the Michaelis–Menten equation (Lorsch, 2014). Kinetic parameters *K*_m_ and *K*_i_ with inhibition were obtained by GraphPad Prism 9.0 using the following formulae:

(1) $$Y=V_{\max}*X/\left(K_{m}+X*\left(1+X/K_{i}\right)\right)$$

(2) $$k_{\mathrm{cat}}={60*V}_{\max}*MW$$

where, X is substrate concentration (µM), Y is enzyme activity (U/µg), *V*_max_ is the maximum reaction velocity in the same units as Y, *K*_m_ is the Michaelis-Menten constant (µM), *K*_i_ is the dissociation constant in the same units as X. MW stands for molecular weight (g/mol), *k*_cat_ is catalytic number, also called turnover number, in the units of s^−1^ (Motulsky, 2023).

### Structural modeling, molecular docking and structural analysis

The theoretical structures of AbXOD and mutants were modeled by Robetta (https://robetta.bakerlab.org). Molecular docking was performed on Molecular Operating Environment (MOE) 2019.0102 and structure analysis was carried out by PyMOL2.2.0. The complex structure of XOD was prepared by preserving the conformation of protein, supplementing incomplete residues, and only set the active site ligand. Then, the holo-structures of XOD complexed with ligands were utilized to define the active/binding sites surrounding the ligands. The parameters were set at the default values in MOE, according to the score order of the combined conformation simulated by the software, the docking model with the largest binding energy was selected as the analysis object. The 2-D ligand interactions including hydrogen bonds were analyzed and plotted by PyMOL.

### Purine reduction by Q201E in fish sauce

Q201E was evaluated by the production of uric acid in fish sauce. Each 600 μL food reaction system consists of 500 μL of fish sauce (PHOENIX&EARTH, China) prepared at pH 9.0, 100 μL of 10 U/mL Q201E, containing potassium oxonate at the final concentration of 0.1 mmol/L. After incubation at 40 °C for 30 min, the reaction solution was heated in boiling water for 20 min to inactivate the Q201E enzyme and to end the reaction. According to our previous study (Zhang et al., 2019), the composition of the reaction mixture was determined by HPLC. The sample was filtered through a 0.22 µm syringe filter (Tianjin Branch billion Lung Experimental Equipment Co., LTD, China), and 10 μL was transferred to a Symmetry Shield RP18 column (100Å, 5 µm, 4.6 mm × 250 mm, 1 pk, Waters). UV absorption spectra at 270 nm were collected. Isocratic HPLC analysis was performed at 25 °C at a flow rate of 0.5 mL/min using a 2695 HPLC system (Waters, America) equipped with a 2998 photodiode array detector (Waters, America). The mobile phase consisted of 95% 20 mmol/L potassium dihydrogen phosphate and 5% methanol, pH 4.1. And standard curve (y=6.326×10^−8^x-0.0115, R^2^=0.999), was built in the concentration range of 0.5, 1, 2, 5 and 10 mmol/L for uric acid which was used to calculate the uric acid content (x) of samples from their peak areas (y). Standard uric acid was purchased from Sigma Company in Germany.

### Statistical analysis

Experimental data were measured in parallel three times and repeated two times. The effects of amino acid substitutions on substrate inhibition and enzyme activity will be discussed based on statistical analyses, focusing on the alteration of the global structure of the enzyme.

## Results

### Saturation mutagenesis at Gln 201

We set out to carry out a detailed saturation mutagenesis investigation of Q201 to hopefully improve the enzyme activity under high substrate concentrations. A total of 96 single colonies were randomly selected and assayed from the site-directed mutagenesis library, which corresponds to more than 95% coverage of all the single mutations. Most of the Q201 substitutions resulted in lower enzyme activity than the wild type at 5 mmol/L xanthine, and even inactivation (Supplementary Fig. 2**A**). The sequencing results showed that the substitution of Gln201 to Ser, Leu, Thr, Val, Pro, and Cys resulted in greater enzyme activity than the wild-type on 5 mmol/L xanthine. However, it is worth noting that all these newly acquired mutations are completely different from the naturally occurring ones at the 201st site in XODs, which are Gly, Gln, Asp, Ala, His, Thr and Glu, as shown in Supplementary Fig. 1.

**Fig. 1:**
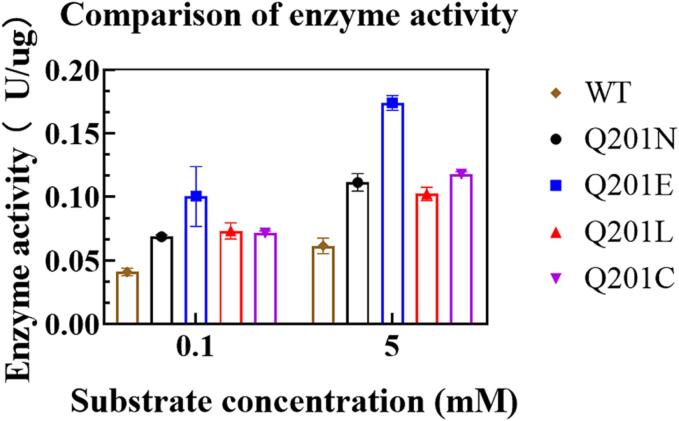
Enzyme activity in two substrate concentrations. Two different xanthine substrate concentration models, commonly used standard substrate concentration model — 0.1 mmol/L and high substrate concentration model —5 mmol/L.

The two best-performing mutants, Q201L and Q201C were further purified and characterized. In addition, to better understand the contribution of Q201 to the substrate inhibition, the two closest amino acids to Gln, Glu and Asn were introduced to make mutants Q201E and Q201N. As shown in Fig. 1, all four mutants showed better catalytic activities in both low substrate concentration (0.1 mmol/L xanthine) and high concentration (5 mmol/L xanthine), and Q201E displayed the highest catalytic activity, nearly 2.9-fold higher than that of the wild-type enzyme on 5 mmol/L xanthine.

**Fig. 2:**
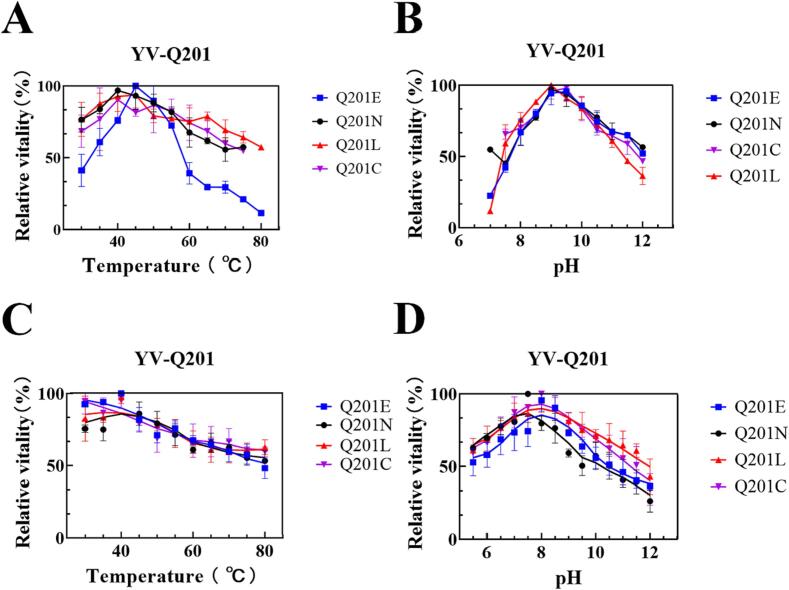
Characterization of the enzymatic properties of the mutants. (A) Temperature-activity profile. (B) pH-activity profile. (C) Temperature-stability profile. (D) pH-stability profile. Enzymatic assays were carried out with 5 mmol/L xanthine.

The effects of temperature and pH on enzyme activity were determined using 5 mmol/L xanthine as a substrate **(**Fig. 2**)**. On the high concentration of xanthine (5 mmol/L), all the four mutants displayed very similar temperature-stability, pH-activity and pH-stability profiles to the wild-type. Although Q201E showed a little higher optimum temperature (45 ^o^C) than Q201N, Q201L and Q201C, which displayed their maximum activities at 40°C, all of them had nearly the same temperature tolerance (Fig. 2**A,** Fig. 2**C**). All four mutants shifted the pH optima to pH 9-9.5, which increased by 2 pH units than the pH 7.0 of the wild-type. Meanwhile, they exhibited good stability under alkaline conditions, and even remained above 50% residual activity after being stored at pH 10 for 14 h. The results are consistent with previous reports (Kheirollahi et al., 2017, Yu et al., 2017), which also indicate that the change of the flexible loop could improve the stability of the enzyme.

As shown in Table 1, all four mutants obviously decreased the apparent *K*_m_ values on substrate xanthine, indicating increased affinity for the substrate (xanthine) (Leimkuhler, Stockert, Igarashi, Nishino, & Hille, 2004). Meanwhile, all mutants, Q201N, Q201E, Q201L and Q201C, slightly increased the *k*_cat_ values by 53.15%, 95.15%, 52.39% and 25.09%, resulting in large increases in the apparent second-order rate constant, *k*_cat_*/K*_m_ values (approximately 4.58-fold for Q201N, 7.36-fold for Q201E, 1.75-fold for Q201C, 2.57-fold for Q201L), respectively. These results suggest that the enzymatic activity was increased by the mutations predominantly due to an effect on the rate-limiting reaction in the kinetic process from the xanthine binding to product release (Harris and Massey, 1997, Ray, 1983).

**Table 1:** Steady-state kinetic parameters for variants.

Variant	*Km* (µmol/L)	*kcat* (s^−1^)	*Ki* (µmol/L)	*kcat*/*Km* (µmol^−1^L s^−1^)	*Ki*/*Km*
Wild-type	13.31	409.65	21.11×10^3^	30.78	1.59×10^3^
Q201N	4.45	627.38	11.15×10^3^	140.98	2.50×10^3^
Q201E	3.53	799.44	NI^a^	226.47	NA^b^
Q201C	11.66	624.25	NI^a^	53.81	NA^b^
Q201L	6.48	512.46	115.9×10^3^	79.08	17.89×10^3^

Kinetic parameters were determined by fitting initial rate data to Michaelis-Menten equations using Graphpad Prism 9.0. All steady-state experiments were performed at 40 ^o^C and pH 8.5.

a NI: Not Inhibited.

b NA: Not Available.

Of interest, Q201E and Q201C totally eliminated the SI phenomenon, and Q201L (115.9×10^3^ µM) also showed increased *Ki* is approximately 5.49-fold to wild-type (21.11×10^3^ µM). While Q201N (11.15×10^3^ µM) showed decreased *Ki* which is 52.81% of the wild-type (21.11×10^3^ µM). However, if we use the ratio of *K*_i_*/K*_m_ to characterize the overall substrate inhibition, Q201L also significantly lowered the substrate inhibition by 11.25-fold to 17.89×10^3^ of *K*_i_*/K*_m_.

(Table 1)

### Structural analysis

**Fig. 3:**
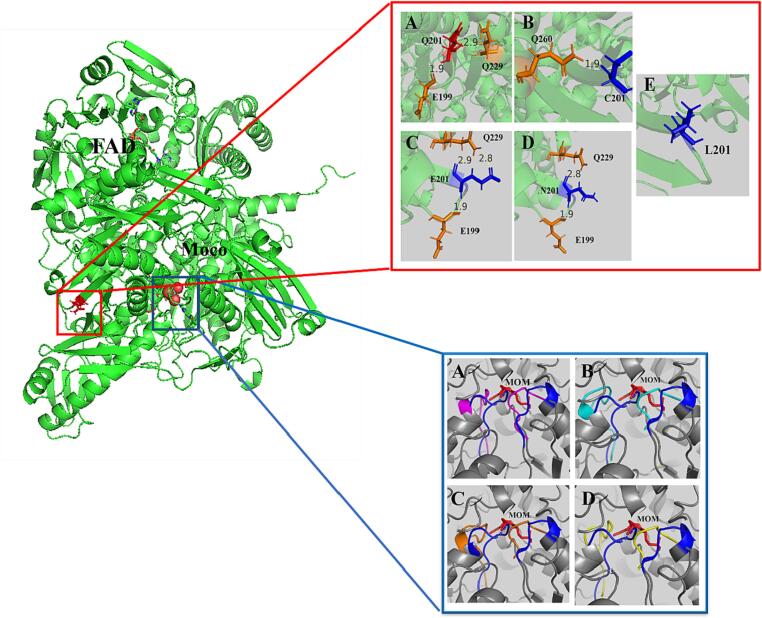
Conformational fluctuations in the predict structures of XOD. The entire model in the green cartoon, molybdopterin structure in red spheres, and Q201 is represented in the red stick. The Q201 mutant interacts with surrounding amino acid hydrogen bonds in the red frame, Q201 in A is shown by red stick, the residue has hydrogen bonding interact with Q201showed by orange stick, and the hydrogen bonding in yellow dotted line. (A)Wide-type (B)Q201C (C) Q201E (D)Q201N (E) Q201L. The conformational changes in the active center in the blue frame, the wild-type show as the blue flexible loop (*loop^234-245^* (Ser234-Arg235-Arg236-Met237-Gly238-Gly239-Gly249-Phe241-Gly242-Lys243-Glu245) and *loop^523-530^* (Ser523-Ala524-Thr525-Ala526-Ala527-Ser528-Ser529-Gly530)), (a) two flexible loops are shown by purple in Q201C; (b) two flexible loops are shown by turquoise blue in Q201E; (c) two flexible loops are shown by orange in Q201N; (d) two flexible loops are shown by yellow in Q201L.

As shown in Fig. 3, the conformation of two flexible loops around the substrate in the active center changed significantly. Meanwhile, the change of local residues can also lead to structural change and the change of enzymatic properties, especially the contribution to stability (Gerlt, Kreevoy, Cleland, & Frey, 1997). As shown in the red box in Fig. 3, the hydrogen bond between Gln201 and Glu199 and Gln229 in the WT was 1.9Å and 2.9 Å, respectively, and in Q201N, exist two hydrogen bonds between N201 and Glu199 and Gln229 were 1.9Å and 2.8 Å. In Q201C, only a 1.9 Å hydrogen bond is connected to Gln260. In addition, Q201E contained three hydrogen bonds between glutamic acid and adjacent amino acids, 1.9 Å, 2.9 Å and 2.8 Å, respectively (one more than the wild type), so it has a more stable α-helix sheet structure (Nelson & Cox, 2008). However, there is no hydrogen bond interaction between Q201L and surrounding amino acids, which makes the local conformation less stable and more flexible.

**Fig. 4:**
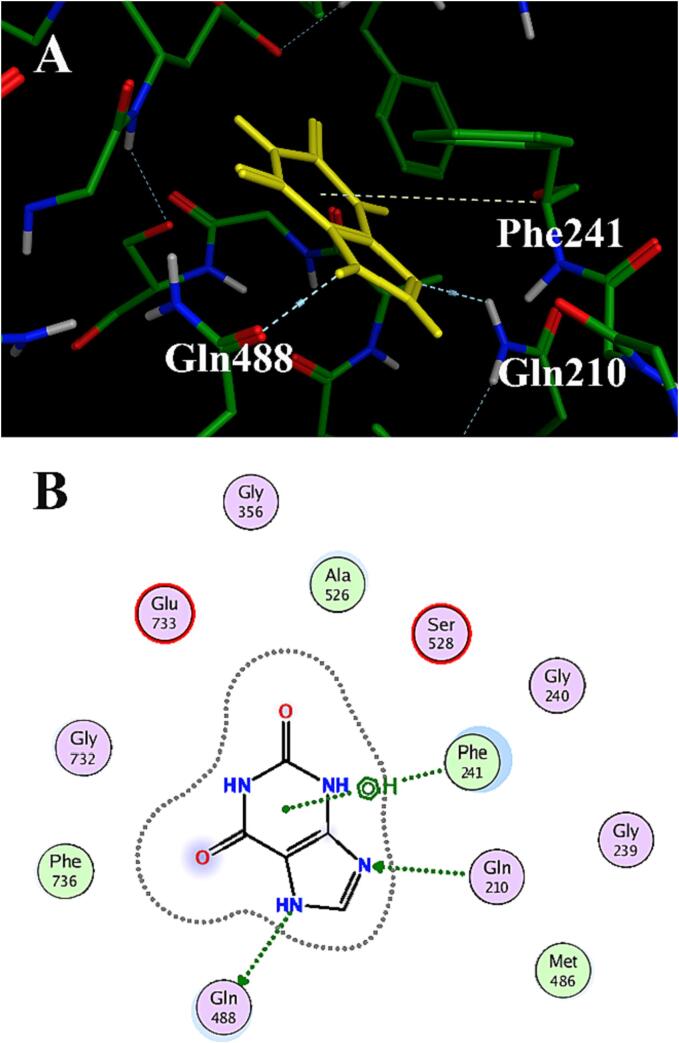
Docking interaction plots of xanthine with flexible loop residues of Q201E. (A) interacting residues of Q201E were shown in sticks colored by atom type, the carbon in green, hydrogen in white, oxygen in red, and nitrogen in blue. Interactions included non-covalent bonds, π interaction and contacts, the hydrogen bonds in blue, and π-cation in yellow. But xanthine is shown in yellow sticks. (B) 2D interaction schematic of Xanthine molecule with Q201E.

Molecular docking was performed to further investigate the effects on enzyme binding to xanthine when structural reorientation in *loop*^234−245^ and *loop*^523−530^ which are located around the active sites of XOD by MOE, and three amino acids interactions were identified which were Gln210, Gln488, and Phe241(Fig. 4**)**. Functionally, Gln210 and Gln488 formed hydrogen bonds with N_7_ and N_9_ on xanthine, stabilized substrates by hydrogen bonding to control substrate entry into the active pocket and facilitate catalytic occurrence (Liu et al., 2021). Simultaneously, Phe241 occupies the aromatic loop of the substrate by π-π stacking interaction.

### Use of Q201E to reduce Hypoxanthine and xanthine in food

**Fig. 5:**
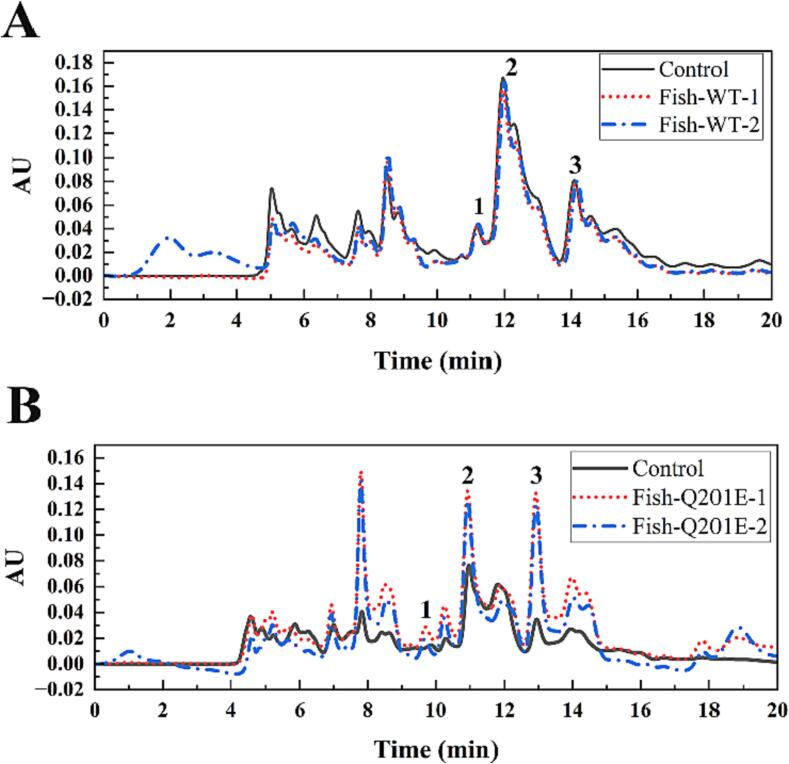
HPLC analysis of uric acid and other purine contents of fish sauce (pH=9.0) with WT (A), and fish sauce (pH=9.0) with Q201E (B). The numbers indicate purine standards, uric acid (1), hypoxanthine, and xanthine. The samples with and without treatment of Q201E (1.6 U/mL) at 40℃ for 30 min are shown in line, short dot, and short dash-dot, respectively. Control, the centrifuged supernatant of Q201E enzyme inactivated in boiling water for 20 min was used to replace the reaction enzyme solution in the enzymatic reaction system.

The application prospect of alkaline Q201E in the production of low-purine food was discussed with fish sauce and fish sauce supplemented with 1 mmol/L xanthine. As shown in Fig. 5**, Q201E** effectively affected the contents of hypoxanthine, xanthine, and uric acid in fish sauce at pH 9.0 and 40℃. Since XOD successively converts hypoxanthine to xanthine and then to uric acid, the uric acid content can be used as an indicator for evaluating XOD. After incubation with 1.6 U/mL Q201E at pH 9.0, 40 °C for 30 min, the uric acid concentration in fish sauce (pH=9.0) increased to 15.81 ± 1.38 µmol/mL, which was 7 folds of wild type (2.26 ± 1.16 µmol/mL). Xanthine concentration increased to 103.09 ± 10.41 µmol/mL, which was 14.6 folds to wild type (7.08 ± 3.58 µmol/mL).

## Discussion

Compared to the wild-type, mutant Q201E showed a decreased *K_m_* of 3.53 µM, indicating an increase in substrate affinity which affects rate-limiting reaction through substrate binding. In addition, the optimal temperature and pH of the mutant Q201E changed significantly to 45℃ and pH 9.0 compared with those of the wild type which were 50℃ and pH 7. In terms of reaction temperature, a lower reaction temperature is beneficial for energy saving and cost reduction in industrial production (Cao et al., 2010, Zhuge et al., 2010). On the other hand, increasing the alkaline tolerance and pH optimum of XOD is more suitable for the reaction in alkaline medium, because both the substrate xanthine and the product uric acid are alkali-soluble substances, and the more alkaline conditions are favorable for higher solubility and higher concentration of substrate. Therefore, the mutant enzyme is more suitable for the reaction under alkaline conditions. Adapting to the condition of the reaction medium is beneficial to relieve substrate inhibition and maximize the use of enzyme activity(Mindell, 2012, Turner, 2010).

In addition, we revealed that distal residues were associated with conformational changes in two loops in active center. Based on these observations, we hypothesized that substrate inhibition might be reduced by changes in protein flexible loops. Homologous sequence comparison of xanthine oxidase shows that the Q201 site is relatively conserved in the secondary structure composed of surrounding residues, which was highly conserved **(**Fig.1**)**. The augment of catalytic activity of the enzyme from conservative over non-conservative changes may have the additional advantages of more stability, more chance of independent synergism in both stability and the diversity of catalytic mechanisms (Gleason, 1992, Maritano et al., 2000, Rodriguez-Larrea et al., 2010). In consideration of maintaining the stability of the structure of the active center, the residue Q201 at the far end of the substrate channel is mutated to achieve the effect of changing the loop structure in the active center. The structure prediction results showed that substituted Q201 could change the structure around the active center of the enzyme, thus affecting the affinity between the substrate and the enzyme. Increasing evidence has shown that distal mutations more than 10 Å away from the active site may significantly affect enzyme activity (X. Wang et al., 2021). Our result is supported by a previous study that Gly121 which 19 Å far from the active site residues in dihydrofolate reductase, was substituted by directed mutagenesis to eight amino acids, and the replacements cause interactions with the large side chains of surrounding residues, resulting in reduced flexibility and enzyme activity (Gekko, Kunori, Takeuchi, Ichihara, & Kodama, 1994).

The more flexible loop is conducive to substrate binding and product release in a high substrate model(Shang et al., 2020), and the structural reorientation around the active center might result in a change in the affinity between the enzyme and the substrate, which was consistent with the experimental results (Table 1). The mechanism underlying the removal of xanthine substrate inhibition in mutant Q201E was explored by molecular docking analysis **(**Fig. 4**)**. The structure of *loop*^234-245^ and *loop*^523-530^ in the Q201E mutant changed when compared with WT. In WT, *loop*^234-245^ (234-SRRMGGGFGKE-245) and *loop*^523-530^ (523-SATAASSG-530) were close to the molybdenum center, while Q201E and other mutants showed a deflection of these two loops. The *loop*^234-245^ and *loop*^523-530^ of AbXOD **(**Fig. 3**)** seem to play a structural rather than catalytic role, and their conformational changes may affect enzyme binds to the substrate (Chen et al., 2015). In WT, Gln210 has a hydrogen bond with C_2_=O of xanthine, while in Q201E, Gln210 forms a hydrogen bond with N_9_ of xanthine, which has been reported to be associated with molybdenum stability (Okamoto, Matsumoto, Hille, Eger, Pai, & Nishino, 2004). Therefore, we speculate that Gln210 plays a significant role in stabilizing and selecting substrates in active pockets. Meanwhile, a hydrogen bond appeared between Gln488 and N_7_ of xanthine, and there was an π-π stacking interaction between Phe241 and xanthine. The formation of these two interactions could further stabilize the substrate, and the double hydrogen bonds formed between Gln210 and Gln488 also further fixed the substrate orientation (Cao, Hall, et al., 2014; Cao et al., 2010). In addition, we also found an interesting phenomenon that neither the substrate xanthine nor the product uric acid had interaction with the C_8_ site in WT, which was also observed on the substrate in Q201E. However, in the molecular docking between Q201E and the product uric acid, we observed hydrogen bonds formed between amino acids on the loop and C_8_=O, which were absent in the wild-type, probably playing roles in differentiating the substrate and product. And the reduction of substrate affinity seems inconsistent with expectation, it is probably because the structural change makes it easier for the substrate to bind and the product to be released in the channel (Yoshino et al., 2015), thus exhibiting decreased apparent Michaelis constant (K_m_ value), i. e., increased substrate affinity. So we boldly speculated that some amino acids on the loop could recognize substrates and products, although we have not proved it through experiments.

Fish sauce is a kind of clarified fermented condiment obtained by natural fermentation with high content of hypoxanthine. Q201E still displayed good conversion efficiency in fish sauce, which could convert purines into uric acid in fish sauce, and the conversion effectiveness of uric acid was approximately 7-fold to WT, and the conversion activity to xanthine was approximately 14.6 folds to WT. The over-accumulation of xanthine is because of AbXOD's efficient transformation on hypoxanthine, which is of endogenous ones of fish sauce and is also probably derived from inosine and other precursor purine nucleosides and purine nucleotides (Litwack, 2022). In addition, product inhibition of XOD also has been reported (Radi, Tan, Prodanov, Evans, & Parks, 1992), but accumulation of uric acid can be eliminated by adding uric acid oxidase in industrial production. In future commercial production, the purines in fish sauce and their derived forms like xanthine or uric acid will probably be eliminated by *in vitro* reconstituted purinolytic enzymes, as demonstrated in our recently published paper (Shi et al., 2022). Some reported that fish sauce is rich in amino acids and small peptides (Bu et al., 2021, Lopetcharat et al., 2001, Wen-yu et al., 2019), and other active components (Pham, Schilling, Yoon, Kamadia, & Marshall, 2008), which may have potential inhibitory effects on the xanthine oxidase activity (Huang et al., 2022, Zhao et al., 2020). So, we suspected that the obtained purine-reducing result of Q201E has been discounted due to the component complexity of the fish sauce system. In addition, 1/3 of uric acid in the human body is metabolized through the intestine, and uric acid cannot enter the blood through the rat intestine (Bronk and Shaw, 1986, Yasujima et al., 2018), oral administration of uric acid in mice did not cause an increase in plasma uric acid even had a protective effect against indomethacin induced intestinal injury in mice (Wada et al., 2022, Yasutake et al., 2017). And it has been reported that the effect of oral hypoxanthine on blood uric acid in the subjects is higher than that of xanthine (Clifford, Riumallo, Young, & Scrimshaw, 1976), so we believe that the effect of uric acid in food on the human body is less than that of hypoxanthine and purine precursors.

## Conclusions

In conclusion, our study clearly showed that single-point mutations near the substrate channel can effectively improve enzyme activity, and can be applied in food with high substrate concentration. Additionally, we have shown that single amino acid substitutions around enzyme access tunnels can eliminate substrate inhibition. The newly obtained single mutants at Q201 site could greatly increase enzyme activity and apparent substrate affinity. The best performing mutant Q201E completely eliminated the substrate inhibition and increased catalytic activity and turnover number (*k*_cat_) by 1.9- and nearly 2-fold (to 799.44 s^−1^), respectively, in comparison to the wild-type. In the salt-rich high-purine fish sauce system, the uric acid conversion effect of Q201E was approximately 7-fold to the wild-type. The computational modeling and simulation results disclosed obvious conformation changes of the flexible loops around the active center of Q201E, which may underlie the improvement of enzyme activity. The way of changing the distal residues outside the active site to indirectly change the conformation of the flexible loops of the active site is instructive and has new guiding significance for the selection principles of XOD mutation sites.

## CRediT authorship contribution statement

**You Wen:** Writing – original draft, Conceptualization, Methodology, Investigation. **Jiahui Xu:** Data curation, Validation, Investigation. **Donglei Pan:** Formal analysis. **Chenghua Wang:** Project administration, Visualization, Investigation.

## Declaration of Competing Interest

The authors declare that they have no known competing financial interests or personal relationships that could have appeared to influence the work reported in this paper.

## Data Availability

Data will be made available on request.

## References

[b0005] Bortolotti M., Polito L., Battelli M.G., Bolognesi A. (2021). Xanthine oxidoreductase: One enzyme for multiple physiological tasks. Redox Biology.

[b0010] Bronk J.R., Shaw M.I. (1986). The transport of uric acid across mouse small intestine in vitro. The Journal of Physiology.

[b0015] Bu Y., Liu Y., Luan H., Zhu W., Li X., Li J. (2021). Characterization and structure-activity relationship of novel umami peptides isolated from Thai fish sauce. Food & Function.

[b0020] Cao H., Hall J., Hille R. (2014). Substrate orientation and specificity in xanthine oxidase: Crystal structures of the enzyme in complex with indole-3-acetaldehyde and guanine. Biochemistry.

[b0025] Cao H., Pauff J.M., Hille R. (2010). Substrate orientation and catalytic specificity in the action of xanthine oxidase: The sequential hydroxylation of hypoxanthine to uric acid. Journal of Biological Chemistry.

[b0030] Cao H., Pauff J.M., Hille R. (2014). X-ray crystal structure of a xanthine oxidase complex with the flavonoid inhibitor quercetin. Journal of Natural Products.

[b0035] Chen X., Wang Y., Ma Z., Li N., Han W., Zhang Q., Cheng J. (2015). Asp141 and the Hydrogen-Bond Chain Asp141-Asn109-Asp33 Are Respectively Essential for GT80 Sialyltransferase Activity and Structural Stability. Biochemistry (Moscow).

[b0040] Clifford A.J., Riumallo J.A., Young V.R., Scrimshaw N.S. (1976). Effect of Oral Purines on Serum and Urinary Uric Acid of Normal, Hyperuricemic and Gouty Humans. Journal of Nutrition.

[b0045] Colette Daubner S., Lanzas R.O. (2018). Pteridines ☆. Reference Module in Biomedical Sciences, Elsevier..

[b0050] Endo A., Irisawa T., Dicks L., Tanasupawat S., Batt C.A., Tortorello M.L. (2014). Encyclopedia of Food Microbiology.

[b0055] Foucher, G., & Faure, S. (2022). What’s gout? *Actualités Pharmaceutiques, 61*(618), 59-61. https://doi.org/https://doi.org/10.1016/j.actpha.2022.07.015. 10.1016/j.actpha.2022.07.011 PMC9465936 36117877

[b0060] Gekko K., Kunori Y., Takeuchi H., Ichihara S., Kodama M. (1994). Point Mutations at Glycine-121 of Escherichia coli Dihydrofolate Reductase: Important Roles of a Flexible Loop in the Stability and Function. The Journal of Biochemistry.

[b0065] Gerlt J.A., Kreevoy M.M., Cleland W.W., Frey P.A. (1997). Understanding enzymic catalysis: The importance of short, strong hydrogen bonds. Chemistry & Biology.

[b0070] Gleason F.K. (1992). Mutation of conserved residues in Escherichia coli thioredoxin: Effects on stability and function. Protein Science.

[b0075] Harris C.M., Massey V. (1997). The oxidative half-reaction of xanthine dehydrogenase with NAD; reaction kinetics and steady-state mechanism. Journal of Biological Chemistry.

[b0080] Hausladen A., Fridovich I. (1993). Excess substrate inhibition of xanthine oxidase: A reexamination. Archives of Biochemistry & Biophysics.

[b0085] Huang X.-N., Zhang Y.-M., Wen Y., Jiang Y., Wang C.-H. (2022). Protease-Catalyzed Rational Synthesis of Uric Acid-Lowering Peptides in Non-aqueous Medium. International Journal of Peptide Research and Therapeutics.

[b0090] Jankowska D.A., Trautwein-Schult A., Cordes A., Bode R., Baronian K., Kunze G. (2015). A novel enzymatic approach in the production of food with low purine content using Arxula adeninivorans endogenous and recombinant purine degradative enzymes. Bioengineered.

[b0095] Kaneko K., Aoyagi Y., Fukuuchi T., Inazawa K., Yamaoka N. (2014). Total Purine and Purine Base Content of Common Foodstuffs for Facilitating Nutritional Therapy for Gout and Hyperuricemia. Biological & Pharmaceutical Bulletin.

[b0100] Kheirollahi, A., Khajeh, K., & Golestani, A. (2017). Rigidifying flexible sites: An approach to improve stability of chondroitinase ABC I. *International Journal of Biological Macromolecules, 97*, 270-278. https://doi.org/https://doi.org/10.1016/j.ijbiomac.2017.01.027. 10.1016/j.ijbiomac.2017.01.027 28082225

[b0105] Kokkonen P., Beier A., Mazurenko S., Damborsky J., Bednar D., Prokop Z. (2021). Substrate inhibition by the blockage of product release and its control by tunnel engineering. RSC Chemical Biology.

[b0110] Kostić D.A., Dimitrijević D.S., Stojanović G.S., Palić I.R., Đorđević A.S., Ickovski J.D. (2015). Xanthine Oxidase: Isolation, Assays of Activity, and Inhibition. Journal of Chemistry.

[b0115] Kuda T., Holzapfel W. (2015). Advances in Fermented Foods and Beverages.

[b0120] Leimkuhler S., Stockert A.L., Igarashi K., Nishino T., Hille R. (2004). The role of active site glutamate residues in catalysis of Rhodobacter capsulatus xanthine dehydrogenase. Journal of Biological Chemistry.

[b0125] Litwack G., Litwack G. (2022). Human Biochemistry.

[b0130] Liu Y., Han C., Lu T., Liu Y., Chen H., Yang C., Li Y. (2021). Investigation of the interaction between Chrysoeriol and xanthine oxidase using computational and in vitro approaches. International Journal of Biological Macromolecules.

[b0135] Lopetcharat K., Choi Y.J., Park J.W., Daeschel M.A. (2001). Fish Sauce Products and Manufacturing: A Review. Food Reviews International.

[b0140] Lorsch J.R., Lorsch J. (2014). Methods in Enzymology.

[b0145] Mahor D., Priyanka A., Prasad G.S., Thakur K.G. (2016). Functional and Structural Characterization of Purine Nucleoside Phosphorylase from Kluyveromyces lactis and Its Potential Applications in Reducing Purine Content in Food. PLoS One1.

[b0150] Maritano D., Accornero P., Bonifaci N., Ponzetto C. (2000). Two mutations affecting conserved residues in the Met receptor operate via different mechanisms. Oncogene.

[b0155] Mindell J.A. (2012). Lysosomal acidification mechanisms. Annual Review of Physiology.

[b0160] Motulsky, H. J. (2023). Equation: Determine the kcat*.* Retrieved from: http://graphpad-prism.cn/guides/prism/9/curve-fitting/reg_kcat.htm Accessed.

[b0165] Nelson D.L., Cox M.M. (2008).

[b0170] Nishino T., Okamoto K., Kawaguchi Y., Hori H., Matsumura T., Eger B.T., Nishino T. (2005). Mechanism of the conversion of xanthine dehydrogenase to xanthine oxidase: Identification of the two cysteine disulfide bonds and crystal structure of a non-convertible rat liver xanthine dehydrogenase mutant. Journal of Biological Chemistry.

[b0175] Okamoto, K., Matsumoto, K., Hille, R., Eger, B. T., Pai, E. F., & Nishino, T. (2004). The crystal structure of xanthine oxidoreductase during catalysis: Implications for reaction mechanism and enzyme inhibition. *Proceedings of the National Academy of Sciences of the United States of America, 101*(21), 7931-7936. https://doi.org/10.1073/pnas.0400973101. 10.1073/pnas.0400973101 PMC419534 15148401

[b0180] Pauff J.M., Zhang J., Bell C.E., Hille R. (2008). Substrate orientation in xanthine oxidase: Crystal structure of enzyme in reaction with 2-hydroxy-6-methylpurine. Journal of Biological Chemistry.

[b0185] Pham A.J., Schilling M.W., Yoon Y., Kamadia V.V., Marshall D.L. (2008). Characterization of fish sauce aroma-impact compounds using GC-MS, SPME-Osme-GCO, and Stevens' power law exponents. Journal of Food Science and Technology.

[b0190] Radi R., Tan S., Prodanov E., Evans R.A., Parks D.A. (1992). Inhibition of xanthine oxidase by uric acid and its influence on superoxide radical production. Biochimica et Biophysica Acta.

[b0195] Ray W.J. (1983). Rate-limiting step: A quantitative definition. Application to steady-state enzymic systems. *Biochemistry*.

[b0200] Reed M.C., Lieb A., Nijhout H.F. (2010). The biological significance of substrate inhibition: A mechanism with diverse functions. BioEssays.

[b0205] Robert X., Gouet P. (2014). Deciphering key features in protein structures with the new ENDscript server. Nucleic Acids Research.

[b0210] Rodriguez-Larrea D., Perez-Jimenez R., Sanchez-Romero I., Delgado-Delgado A., Fernandez J.M., Sanchez-Ruiz J.M. (2010). Role of conservative mutations in protein multi-property adaptation. The Biochemical journal.

[b0215] Rubbo H., Radi R., Prodanov E. (1991). Substrate inhibition of xanthine oxidase and its influence on superoxide radical production. Biochimica et Biophysica Acta.

[b0220] Shang Y.P., Chen Q., Li A.T., Quan S., Xu J.H., Yu H.L. (2020). Attenuated substrate inhibition of a haloketone reductase via structure-guided loop engineering. Journal of Biotechnology.

[b0225] Shi P., Zhang R., Liu C.X., Wu S.X., Pei X.D., Jiang Y., Wang C.H. (2022). Computer-assisted in vitro reconstitution of purine degradation pathway to lower the purine content in food. Journal of the Science of Food and Agriculture.

[b0230] Smith P.K., Krohn R.I., Hermanson G.T., Mallia A.K., Gartner F.H., Provenzano M.D., Klenk D.C. (1985). Measurement of protein using bicinchoninic acid. Analytical Biochemistry.

[b0235] Torrelo G., Hanefeld U., Hollmann F. (2014). Biocatalysis. Catalysis Letters.

[b0240] Trautwein-Schult A., Jankowska D., Cordes A., Hoferichter P., Klein C., Matros A., Kunze G. (2013). Arxula adeninivorans Recombinant Urate Oxidase and Its Application in the Production of Food with Low Uric Acid Content. Microbial Physiology.

[b0245] Turner B.L. (2010). Variation in pH optima of hydrolytic enzyme activities in tropical rain forest soils. Applied and Environmental Microbiology.

[b0250] Wada A., Higashiyama M., Kurihara C., Ito S., Tanemoto R., Mizoguchi A., Hokari R. (2022). Protective Effect of Luminal Uric Acid Against Indomethacin-Induced Enteropathy: Role of Antioxidant Effect and Gut Microbiota. Digestive Diseases and Sciences.

[b0255] Wang C.H., Zhao T.X., Li M., Zhang C., Xing X.H. (2016). Characterization of a novel Acinetobacter baumannii xanthine dehydrogenase expressed in Escherichia coli. Biotechnology Letters.

[b0260] Wang X., Zhang X., Peng C., Shi Y., Li H., Xu Z., Zhu W. (2021). D3DistalMutation: A Database to Explore the Effect of Distal Mutations on Enzyme Activity. Journal of Chemical Information and Modeling.

[b0265] Wen-yu G., Jin-ling S., Hua-rong G., Hong-tao M., Xiang-yang G. (2019). Study on the Taste Characteristics of Gly-Pro and Val-Hyp in Fish Sauce. Science and Technology of Food Industry.

[b0270] Wu B. (2011). Substrate inhibition kinetics in drug metabolism reactions. Drug Metabolism Reviews.

[b0275] Yasujima T., Murata C., Mimura Y., Murata T., Ohkubo M., Ohta K., Yuasa H. (2018). Urate transport function of rat sodium-dependent nucleobase transporter 1. Physiological Reports.

[b0280] Yasutake Y., Tomita K., Higashiyama M., Furuhashi H., Shirakabe K., Takajo T., Hokari R. (2017). Uric acid ameliorates indomethacin-induced enteropathy in mice through its antioxidant activity. Journal of Gastroenterology and Hepatology.

[b0285] Yoshino M., Murakami K. (2015). Analysis of the substrate inhibition of complete and partial types. Springerplus.

[b0290] Yu H., Yan Y., Zhang C., Dalby P.A. (2017). Two strategies to engineer flexible loops for improved enzyme thermostability. Scientific reports.

[b0295] Zhang R., Gao S.-J., Zhu C.-Y., Sun Y., Liu X.-L., Zhao M.-M., Wang C.-H. (2019). Characterization of a novel alkaline Arxula adeninivorans urate oxidase expressed in Escherichia coli and its application in reducing uric acid content of food. Food Chemistry.

[b0300] Zhao J., Huang L., Sun C., Zhao D., Tang H. (2020). Studies on the structure-activity relationship and interaction mechanism of flavonoids and xanthine oxidase through enzyme kinetics, spectroscopy methods and molecular simulations. Food Chemistry.

[b0305] Zhuge B., Xu Q., Du G., Zhang M., Fang H., Zhuge J. (2010). Properties and Application of Cold-adapted Enzymes Produced by a Psychrotrophic Bacterium. Chinese Journal of Applied and Environmental Biology.

