# Supplementary material for: Removal of substrate inhibition of Acinetobacter baumannii xanthine oxidase by point mutation at Gln-201 enables efficient reduction of purine content in fish sauce
Source: Food Chem X. 2023 Feb 4;17:100593. doi: 10.1016/j.fochx.2023.100593 (PMC9944496; doi:10.1016/j.fochx.2023.100593)

**Removal of substrate inhibition of *Acinetobacter baumannii* xanthine oxidase by point mutation at Gln-201 enables efficient reduction of purine content in fish sauce**

You WEN^a^, Jiahui XU^a^, Donglei PAN^a^, Cheng-Hua WANG^a,^*

^a^ College of Light Industry and Food Engineering, Guangxi University, Nanning 530004, People’s Republic of China

You WEN, E-mail: [weny97@163.com](mailto:weny97@163.com);

Jiahui XU, E-mail: [1412021957@qq.com](mailto:1412021957@qq.com);

Donglei PAN, E-mail: [1016074587@qq.com](mailto:1016074587@qq.com);

Chenghua WANG, E-mail: [chwang@gxu.edu.cn](mailto:chwang@gxu.edu.cn)

*Corresponding author.

Cheng-Hua Wang, Address: College of Light Industry and Food Engineering, 100 Daxue East Road, Nanning 530004, People’s Republic of China. Tel: +86-771-323-2874, Fax: +86-771-323-2874, E-mail: chwang@gxu.edu.cn;

**Supplementary Figure 1** **Alignment of the N-terminal segment of representative XOD sequences.**


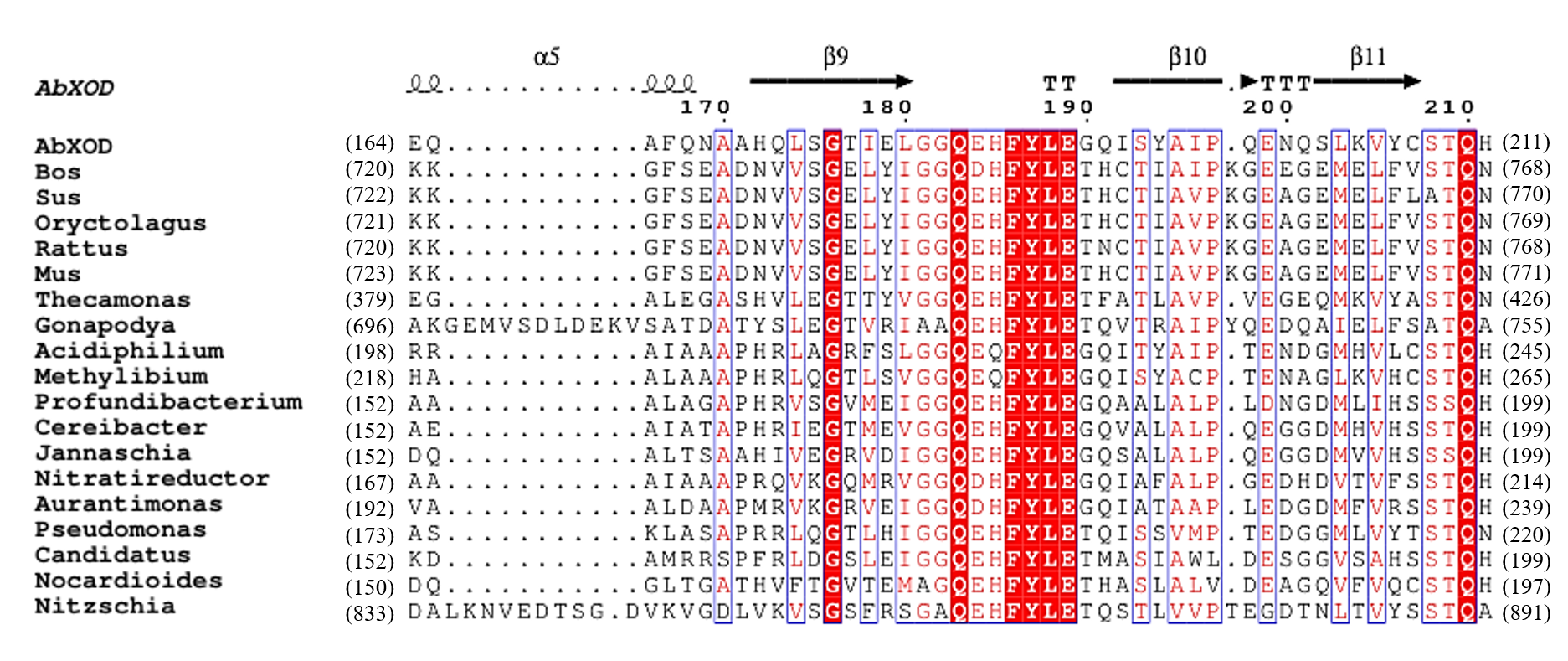


**Supplementary Figure 2 Enzyme activity in two substrate concentrations.**


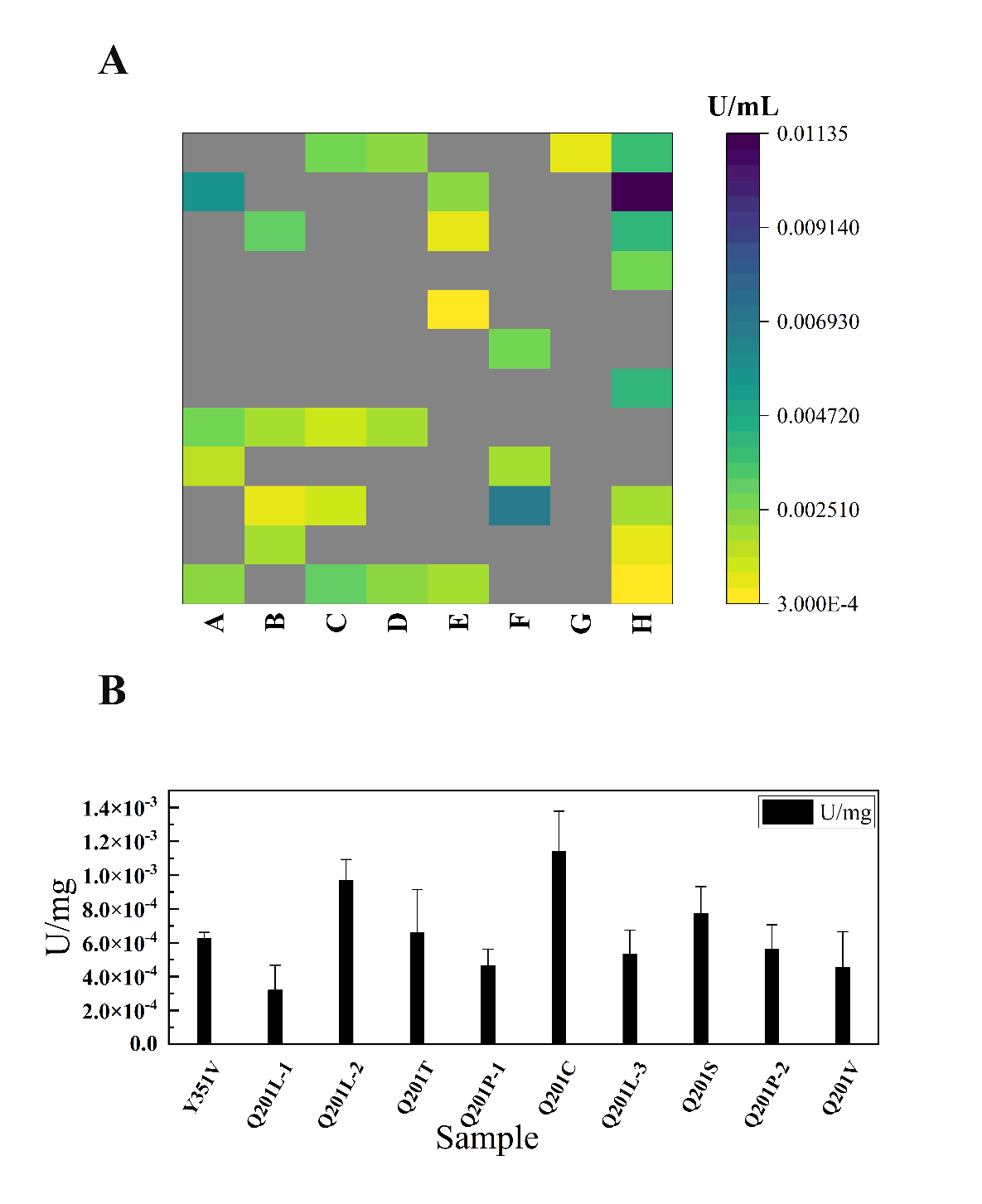

Supplement: Supplementary data 1 [file mmc1.docx]
